# Unraveling the genetic diversity and structure of *Quercus liaotungensis* population through analysis of microsatellite markers

**DOI:** 10.7717/peerj.10922

**Published:** 2021-04-14

**Authors:** Bin Guo, Xiangchun Hao, Lijun Han, Yu Zhai, Shuai Zhou, Si Chen, Da Ren, Xinmin An

**Affiliations:** 1 Beijing Forestry University, Beijing, China; 2 Shanxi Academy of Forestry and Grassland Sciences, Taiyuan, China

**Keywords:** Quercus liaotungensis Koidz., Genetic diversity, Genetic structure, Microsatellite marker

## Abstract

**Background:**

*Quercus liaotungensis* Koidz. is an ecologically and economically important tree species widely distributed in Northern China. However, the effective assessment, utilization, and protection of *Q. liaotungensis* resources remain unexplored.

**Methods:**

In total, 120 samples obtained from 12 *Q. liaotungensis* populations of Northern China were investigated for genetic diversity and structure using 19 simple sequence repeat (SSR) primer pairs.

**Results:**

The total number of alleles detected was 293, the average number of effective allele (*N*e) was 6.084, the genetic differentiation coefficient (*F*st) was 0.033, and the mean observed heterozygosity (*H*o) and expected heterozygosity (*H*e) were 0.690 and 0.801, respectively. Moreover, analysis of molecular variance (AMOVA) showed a 5.5% genetic variation among 12 *Q. liaotungensis* populations, indicating that a high level of genetic diversity and a low degree of genetic differentiation among *Q. liaotungensis* populations. STRUCTURE and cluster analysis divided the 12 *Q. liaotungensis* populations into the following three subpopulations: Bashang Plateau subpopulation (SH), Liaodong Peninsula subpopulation (NC), and Loess Plateau subpopulation (other 10 populations). The cluster analysis based on 19 climatic factors was consistent with the genetic structure. A positive correlation was found between genetic distance and geographical distance (*r* = 0.638, *p* = 0.028) by the Mantel test, and two boundaries were found among the 12* Q. liaotungensis* populations by the Barrier analysis, indicating that *Q. liaotungensis* populations existed isolated by geographical distance and physical barrier.

**Conclusion:**

This study suggests that geographical isolation, physical barrier, climatic types, and natural hybridization promote the formation of genetic structures, which can contribute to future protection and genetic improvement of *Q. liaotungensis.*

## Introduction

*Quercus liaotungensis* Koidz. is a dominant species found in deciduous broad-leaved forests of Northern China (Sun et al., 2018) with significant ecological and economic value (Yu, Zhou & Luo, 2003). The timber of *Q. liaotungensis* can be used to prepare building materials (Xu et al., 2018). In addition, it has a range of other functions, including the source of cultivation media for edible fungus, multiple nutrients, and medical materials, and carbon fixation, carbon sequestration, and soil and water conservation (Du et al., 2007; Li et al., 2012; Wang & Zhang, 2011). In the early decades of the 20th century, the natural forests of *Q. liaotungensis* constituted the indigenous vegetation of the Loess Plateau of China (Du et al., 2007). However, this species has rapidly dwindled due to extensive human disturbances and climatic changes and currently exists in the form of secondary forests with patchy distribution (Hou et al., 2004; Li & Ma, 2003). Moreover, the taxonomic position of two closely related species, i.e., *Q. liaotungensis* and *Q. mongolica,* is unclear. Although frequent gene exchange can cause natural hybridization between them, they should be considered as discretely taxonomic units according to the studies by Zeng et al. (Wei et al., 2015; Zeng et al., 2010; Zeng et al., 2011).

The genetic diversity of a species affects its adaptability to the environment and the stability of the forest ecosystem (Hughes et al., 2008), including various traits of economic significance present in forest trees, such as the size of stem, wood quality, drought resistance, and disease resistance. Therefore, analyzing the genetic diversity among and within populations is important to evaluate evolution and the potential for improvement of tree species. In recent years, several studies have reported the genetic diversity and structures of Quercus, such as *Q. acutissima* (Zhang et al., 2013), *Q. ilex* (Fernádez i Marti et al., 2018), *Q. variabilis* (Shi et al., 2017), *Q. mongolica* (Ueno & Tsumura, 2008), and *Q. rubra* (Pettenkofer et al., 2019). At present, the research on *Q. liaotungensis* mainly focuses on its ecological function (Sun et al., 2019), distribution pattern (Li et al., 2012; Wu, Zheng & Ma, 2002), natural regeneration (Sun, Gao & Chen, 2004; Zhang, Deng & Guan, 2014), and dispersal pattern of acorns (Wang, Ma & Liu, 1999; Zhang, Chen & Zhang, 2008). However, there are a few studies on the genetic diversity of *Q. liaotungensis* populations. Only Qin studied the genetic diversity of eight natural *Q. liaotungensis* populations from the Shanxi Province using 11 SSR primer pairs (Qin et al., 2012), and Wang et al. (2014) studied the genetic diversity of 8 *Q. liaotungensis* populations from the Shanxi Province at different altitudes using SRAP markers. The sampling strategy in a similar distribution incompletely assessed the genetic diversity of *Q. liaotungensis* with previous studies. Besides, climatic conditions not only promote genetic diversity but also play an essential role in the formation of the genetic structure of the *Q. liaotungensis* population. Furthermore, studies on genetic patterns and demographical histories of *Q. liaotungensis* suggest that soil and climatic conditions are the primary factors affecting the genetic diversity of the tree species (Yang et al., 2018).

Simple Sequence Repeat (SSR) markers are highly polymorphic, stable, codominant, and hence regarded as effective means to study the genetic diversity and genetic structure of Quercus species (Fernádez i Marti et al., 2018; Sullivan et al., 2013; Zhang et al., 2013). Zeng et al. (2010) assessed the taxonomic status of two closely related Chinese oaks using SSR and amplified fragment length polymorphism (AFLP) markers in their early reports. Subsequently, nuclear and chloroplast markers were used to analyze the demographical histories in the hybrid zones of *Q. liaotungensi*s and *Q. mongolica* (Zeng et al., 2011). Yang et al. (2016) found that their interspecific divergence and phylogeographical histories were possibly related to the climatic events of the Pleistocene era by studying the four chloroplast DNA and two nuclear genes. Therefore, the collection of sufficient samples from different natural distributions, exploitation of accurate detection, and selection of sufficient markers are necessary to study the genetic pattern of *Q. liaotungensis* species.

Here, we used 19 SSR primers to detect and estimate the genetic variation of *Q. liaotungensis* from different distribution zones in China. The objectives of this study are as follows: (1) how the genetic diversity and genetic differentiation among 12 *Q. liaotungensis* populations; (2) the genetic structure and the number of subpopulations were assessed; (3) whether the geographical distance, physical barrier, and climate factors influenced the genetic structure. These findings are advantageous for revealing genetic information and assisting the conservation and utilization of *Q. liaotungensis* germplasm resources.

## Materials & Methods

### Plant materials

**Figure 1:**
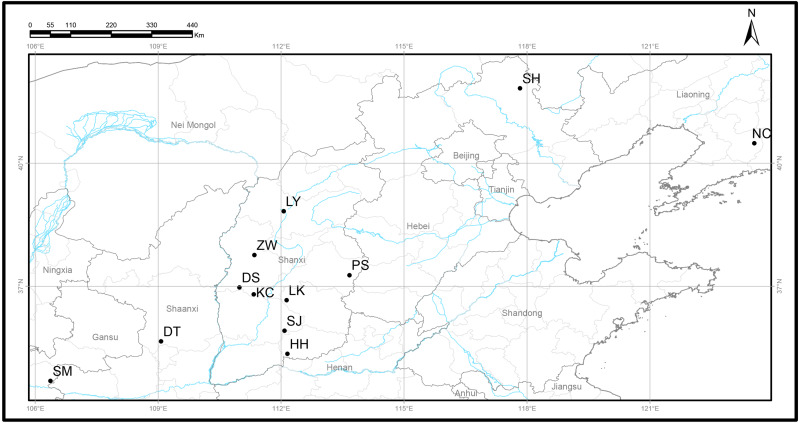
Locations of 12 natural *Q. liaotungensis* populations in China. Map credit: OpenStreetMaps.

A total of 120 *Q. liaotungensis* samples from 12 natural populations were collected from five provinces in China (Fig. 1, Table 1), and studies were conducted on 10 individuals from each population. *Q. liaotungensis* is mainly distributed in the Loess Plateau (Shanxi, Shannxi, Gansu Province) of Northern China. Bashang Plateau (Hebei Province) and Liaodong Peninsula (Liaoning Provinces) are present in the distribution on an overlapping ecological niche of *Q. liaotungensis* and *Q. mongolica* species. The distance of each individual was above 50 m. Young leaves were collected and dried using silica gel for DNA extraction and stored at −80 °C until use.

### DNA extraction and PCR amplification

The total genomic DNA was extracted from young leaves using the DNA secure Plant Kit (Tiangen Biotech, Beijing, China). The quality and concentration of DNA were detected by agarose gel electrophoresis and NanoDrop 2000 spectrophotometer (Implen, CA, USA), respectively. High-quality DNA was used immediately or stored at −20 °C until use.

The genetic diversity of 12 *Q. liaotungensis* populations was studied using 19 SSR primers (Aldrich et al., 2002; Dow, Ashley & Howe, 1995; Kampfer et al., 1998; Mishima et al., 2006; Steinkellner et al., 1997; Wang, 2012) (Table 2). Amplification was performed in a 10 µL reaction mixture, containing 1 µL of DNA template (10 ng/µL), 5 µL of 2 × Taq PCR Master Mix (TaKaRa, Japan), 3 µL of ddH_2_O, 0.5 µL of reverse primer(1 µM), and 0.5 µL of forward primer (1 µM) with a fluorescent label with FAM, HEX (Sangon, Shanghai, China). The PCR program was as follows: pre-denaturation at 94 °C for 5 min, followed by 35 cycles of denaturation at 94 °C for 30 s, annealing at 55 °C for 30 s, and extension at 72 °C for 30 s, with a final extension at 72 °C for 10 min. The PCR products were analyzed by capillary electrophoresis in an ABI 3730xl DNA Analyzer (Applied Biosystems, Foster City, CA, USA). The SSR allele size was determined by GeneMarker version 2.2 software (Genetics, State College, PA, USA).

**Table 1:** Geographical information of 12 natural *Q. liaotungensis* populations in China.

Populations	ID	Sample size	Longitude (°E)	Latitude (°N)	Elevation (m)
Pingsong, Shanxi	PS	10	113.67	37.27	1,250–1,361
Luyashan, Shanxi	LY	10	112.06	38.83	1,593–1,743
Kangcheng, Shanxi	KC	10	111.33	36.81	1,109–1,195
Henghe, Shanxi	HH	10	112.15	35.36	1,048–1,277
Linkongshan, Shanxi	LK	10	112.13	36.66	1,182–1,302
Dongshan, Shanxi	DS	10	110.98	36.97	1,383–1,494
Sanjiao, Shanxi	SJ	10	112.08	35.92	1,085–1,146
Zhenwushan, Shanxi	ZW	10	111.35	37.77	1,352–1,434
Shanmen, Gansu	SM	10	106.37	34.69	1,625–1,725
Siheyong, Hebei	SH	10	117.83	41.83	1,025–1,280
Diantou, Shaanxi	DT	10	109.07	35.66	968–1,051
Nancigou, Liaoning	NC	10	123.54	40.49	118–224

**Table 2:** Genetic diversity among 19 simple sequence repeat (SSR) loci of 12 *Q. liaotungensis* populations.

Locus	Repeat motif	Size range (bp)	*N*a	*N*e	*I*	*H*o	*H*e	*F*st	*N*m	*PIC*	Label^a^	Source
bcqm41	(GT)_30_(AT)_2_	123–177	21	7.010	2.334	0.840	0.857	0.042	5.702	0.959	FAM	Mishima et al. (2006)
bcqm42	(GT)_11_	128–154	10	5.102	1.862	0.795	0.804	0.072	3.222	0.929	FAM	Mishima et al. (2006)
bcqm50	(AC)_8_	113–125	7	2.989	1.330	0.571	0.666	0.018	13.639	0.893	FAM	Mishima et al. (2006)
bcqm74	(TG)_10_	141–175	12	6.245	2.065	0.517	0.840	0.020	12.250	0.932	HEX	Mishima et al. (2006)
bcqm76	(GT)_7_	182–222	17	7.051	2.196	0.420	0.858	0.021	11.655	0.951	HEX	Mishima et al. (2006)
bcqm94	(AC)_9_	142–160	10	3.882	1.596	0.782	0.742	0.040	6.000	0.922	FAM	Mishima et al. (2006)
bcqm96	(AGT)_2_(GT)_8_	115–139	12	3.277	1.504	0.698	0.695	0.008	31.000	0.931	FAM	Mishima et al. (2006)
bcqm325	(AC)_10_	104–122	10	4.090	1.696	0.563	0.756	0.009	27.528	0.922	FAM	Mishima et al. (2006)
MSQ4	(AG)_12_	213–255	17	7.225	2.296	0.523	0.862	0.020	12.250	0.963	HEX	Dow, Ashley & Howe (1995)
MSQ13	(GA)_29_	213–259	14	4.568	1.801	0.725	0.781	0.027	9.009	0.940	HEX	Dow, Ashley & Howe (1995)
ssrQrZAG7	(TC)_17_	132–172	19	11.470	2.610	0.775	0.913	0.015	16.417	0.956	FAM	Kampfer et al. (1998)
ssrQrZAG87	(TC)_20_	117–175	20	4.443	2.027	0.633	0.775	0.036	6.694	0.935	FAM	Kampfer et al. (1998)
ssrQrZAG96	(TC)_20_	157–213	22	12.961	2.750	0.808	0.923	0.013	18.981	0.961	HEX	Kampfer et al. (1998)
ssrQrZAG101	(TC)_20_(AG)_15_	153–191	18	3.008	1.680	0.558	0.668	0.050	4.750	0.963	FAM	Kampfer et al. (1998)
ssrQrZAG112	(GA)_32_	88–126	15	5.998	2.043	0.850	0.833	0.077	2.997	0.945	FAM	Kampfer et al. (1998)
ssrQpZAG36	(AG)_19_	218–256	14	3.878	1.710	0.583	0.742	0.056	4.214	0.939	HEX	Steinkellner et al. (1997)
quru-GA-0C11	(GA)_15_	214–250	20	8.549	2.431	0.833	0.883	0.032	7.563	0.957	HEX	Aldrich et al. (2002)
Qden03032	(CT)_12_	402–448	16	3.582	1.792	0.729	0.721	0.046	5.185	0.949	HEX	Wang (2012)
Qden05011	(CT)_8_	177–217	19	10.271	2.529	0.900	0.903	0.016	15.375	0.956	HEX	Wang (2012)
Mean			15.421	6.084	2.013	0.690	0.801	0.033	11.286	0.942		

**Notes.**

*N*a number of alleles *N*e effect number of alleles *I* Shannon’s information index *H*o observed heterozygosity *H*e expected heterozygosity *F*st genetic differentiation coefficient *N*m gene flow *PIC* polymorphism information content

a Forward primers were modified at the 5′ end with a fluorescent label: HEX (green), FAM (blue) (see Materials and Methods, PCR amplification).

### Data analysis

POPGENE version 1.32 software (Yeh et al., 1997) was used to calculate the genetic diversity parameters, such as the number of alleles (*N*a), the effective number of alleles (*N*e), observed heterozygosity (*H*o), expected heterozygosity (*H*e), and Shannon’s information index (*I*). Deviation from Hardy-Weinberg proportions (HWP) was investigated in each population at a single locus (Excoffier & Lischer, 2010), and genotyping errors were screened using Micro-checker software 2.2.3 (Van et al., 2004). Polymorphic information content (*PIC*) at each locus was calculated according to the following formula Eq. (1):

(1) \begin{eqnarray*}\mathrm{PIC}=1-\sum _{i=1}^{n}{P}_{i}^{2}-\sum _{i=1}^{n-1}\sum _{j=i+1}^{n}2{P}_{i}^{2}{P}_{j}^{2}\end{eqnarray*}

(Botstein et al., 1980), where P is the frequency of the jth allele for the ith marker summed over “n” alleles. The genetic differentiation coefficient (*F*st) of each locus and the matrix among 12 *Q. liaotungensis* populations were calculated using FSTAT version2.9.3 software (Goudet, 2001). Gene flow *(N*m) was evaluated using the following formula: *N*m = (1 − *F*st)/4*F*st. Analysis of molecular variance (AMOVA) was performed among and within populations using GenAlEx version 6.5 software (Peakall & Smouse, 2012). A dendrogram was constructed using an unweighted pair-group method with arithmetic means (UPGMA) algorithm based on Nei’s genetic distance among populations using the NTSYS version 2.1 software (Rohlf, 2000). The correlation between genetic distance and geographical distance was detected using the Mantel test (Manel et al., 2003) with 1,000 permutations using GenAlEx software. To test the effects of the physical barrier among 12 *Q. liaotungensis* populations, the geographical location of each population was investigated by space Monmonier’s maximum difference algorithm (Monmonier, 1973) using Barrier version 2.2 (Manni et al., 2004).

Climatic factors from 12 regions of *Q. liaotungensis* populations were obtained from the WorldClim database (https://www.worldclim.org/data/worldclim21.html) at 10 arc-min resolution using ArcGIS software version10.2. The clustering heat map of 19 climatic factors was drawn using Origin 2018 software. The correlation between genetic distance and climatic factors was detected by the Mantel test (Manel et al., 2003) with 1,000 permutations using GenAlEx software.

The clustering of genetic structure was performed using a Bayesian model with the STRUCTURE software package version 2.3.4 (Pritchard & Stephens, 2000). The admixture model was used to perform a Markov chain Monte Carlo (MCMC) simulation algorithm. The membership of each sample was run for a range of subpopulations from *K* = 1 to 10 (the calculation of each *K* value was repeated 10 times), and also for the length of the burn-in period and MCMC after setting the length to 100,000 and 200,000, respectively. The optimal *K* value was selected according to the delta*K* criterion using the “STRUCTURE HARVESTER” online program with the method from Evanno (Earl & Bridgett, 2012; Evanno, Regnaut & Goudet, 2005). Analysis of each sample was performed with CLUMPP software version1.1.2 (Jakobsson & Rosenberg, 2007), and the relative proportions of the cluster in each population were plotted by the Arc-GIS software using geographical distribution diagram.

## Results

### Genetic diversity

We used 19 pairs of SSR primers to generate 293 alleles from 120 individuals of 12 *Q. liaotungensis* populations (Table 2). The results showed an average value of 15.421 for all alleles ranging from 7.000 (bcqm50) to 22.000 (ssrQrZAG96). The effective number of alleles (*N*e) for each locus ranged from 2.989 (bcqm50) to 12.961 (ssrQrZAG96), with an average of 6.084. The observed heterozygosity (*H*o) for each locus varied from 0.420 (bcqm76) to 0.900 (Qden05011), with an average of 0.690, whereas the expected heterozygosity (*H*e) ranged from 0.666 (bcqm50) to 0.923 (ssrQrZAG96), with an average of 0.801. Shannon’s information index (*I*) ranged from 1.330 (bcqm50) to 2.750 (ssrQrZAG96), with an average of 2.013. The genetic differentiation coefficient (*F*st) (0.008(bcqm96)–0.077(ssrQrZAG112)) and polymorphic information content (*PIC*) (0.893(bcqm50)–0.963(ssrQrZAG101)) were detected at all loci. Gene flow (*N*m) varied from 2.997 in locus ssrQrZAG112 to 31.000 in locus bcqm96, with an average of 11.286. 19 loci were accorded with Hardy-Weinberg proportions(HWP).

The genetic diversity of 12 *Q. liaotungensis* populations was shown in Table 3. The average number of alleles (*N*a) was 6.781, and it varied from 6.316 for the SH population to 7.474 for the NC population, whereas the average effective number of alleles (*N*e) was 4.477, ranging from 3.819 for the SH population to 5.224 for the NC population. Shannon’s information index (*I*) varied from 1.498 for the SH population to 1.753 for the NC population, with a mean of 1.599. Mean value of observed heterozygosity (*H*o = 0.688) were less than that of expected heterozygosity (*H*e = 0.737). The mean value of fixation index (*F*is) was 0.067, ranging from 0.062 for the DT population to 0.324 for the SH population, and *Q. liaotungensis* populations accorded with HWP, except for the SH population. Among them, the NC population had the highest genetic diversity parameters (*N*a, *N*e, *H*e, and *I*), whereas the SH population had the lowest. Thus, by comparison, the NC population showed the highest level of genetic diversity.

**Table 3:** Genetic diversity analysis of 12 *Q. liaotungensis* populations using 19 SSR markers.

Population	*N*a	*N*e	*I*	*H*o	*H*e	*F*is
PS	7.000	4.629	1.636	0.732	0.749	0.067
HH	7.053	4.594	1.631	0.687	0.743	0.096
LY	7.316	4.770	1.695	0.712	0.764	0.093
SJ	6.579	4.486	1.561	0.650	0.726	0.145
KC	6.368	4.268	1.522	0.705	0.713	0.041
LK	6.368	4.247	1.534	0.664	0.718	0.069
DS	6.421	4.322	1.561	0.689	0.729	0.104
SM	6.842	4.488	1.583	0.625	0.724	0.171
ZW	7.000	4.637	1.647	0.632	0.749	0.193
DT	6.632	4.243	1.570	0.702	0.725	0.062
NC	7.474	5.224	1.753	0.726	0.786	0.083
SH	6.316	3.819	1.498	0.737	0.714	0.324^a^
Mean	6.781	4.477	1.599	0.688	0.737	0.067

**Notes.**

*N*a number of alleles *N*e effect number of alleles *I* Shannon’s information index *H*o observed heterozygosity *H*e expected heterozygosity *F*is fixation index

a Significant deviation from Hardy-Weinberg proportions (*α* = 0.05).

### Genetic differentiation

The genetic differentiation was low and moderate among populations when the *F*st value was between 0.00–0.05 and 0.05–0.15, respectively, and the *F*st value more than 0.15 showed a high genetic differentiation (Wright, 1965). The genetic differentiation detected at the species level by FSTAT among 12 *Q. liaotungensis* populations was low (*F*st = 0.033, *p* < 0.05), the average gene flow (Nm) was 11.286, which showed that the gene frequently changed among the 12 *Q. liaotungensis* populations. The relatively high level of gene flow was consistent with the low level of genetic differentiation. AMOVA showed that 94.5% of the genetic variation was found within 12 *Q. liaotungensis* populations, and only 5.5% of the variation occurred among 12 *Q. liaotungensis* populations (Table S1). The result was consistent with that of *F*st, implying that the genetic differentiation of *Q. liaotungensis* mainly existed within populations. The matrix of *F*st was estimated between two populations (Table S3), and the results showed that the genetic differentiation coefficient was the largest (*F*st = 0.133) between KC and SH populations.

### Genetic structure of the population

**Figure 2:**
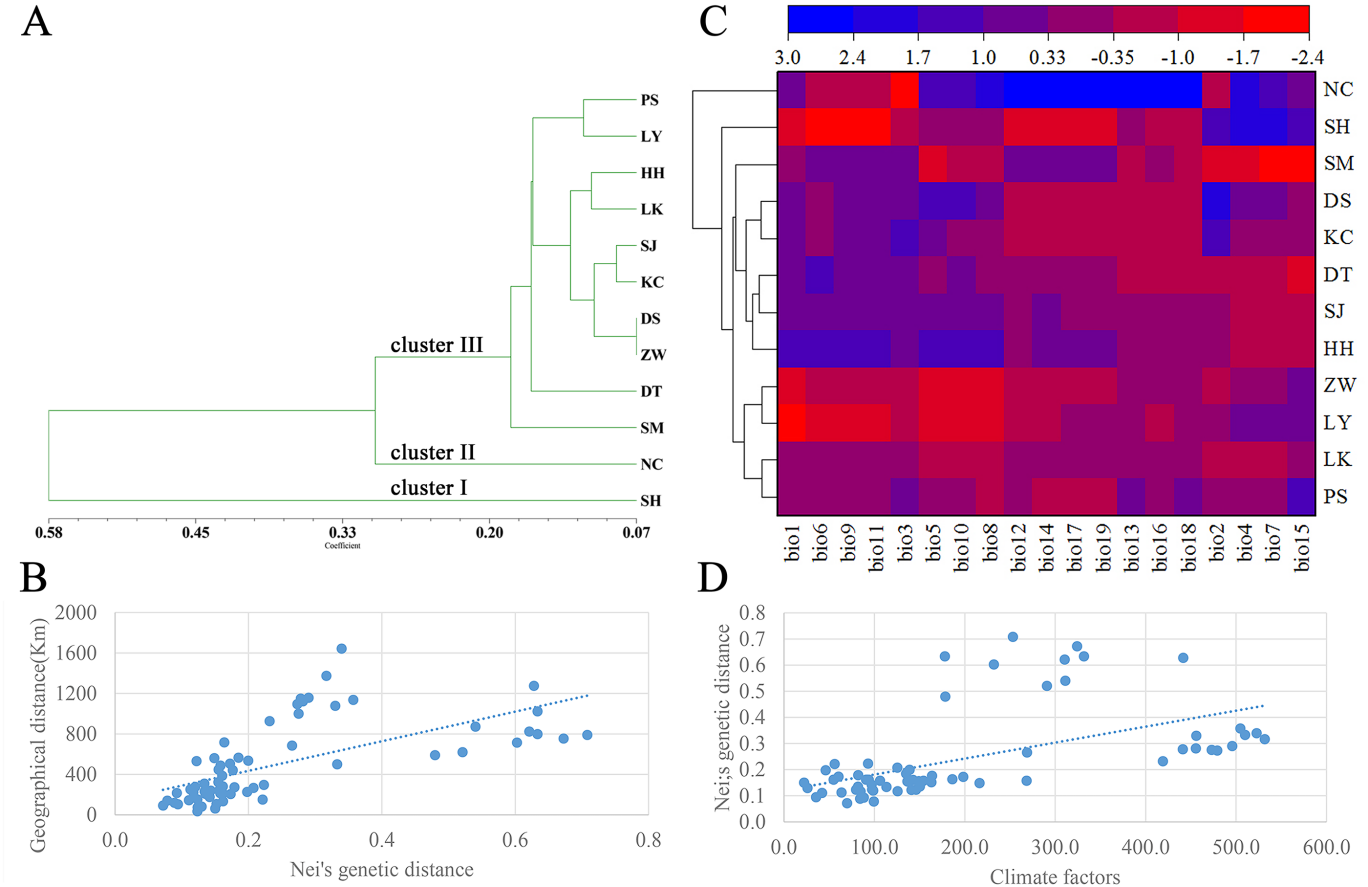
Genetic structure of the population. (A) Cluster analysis of 12 *Q. liaotungensis* populations based on Nei’s genetic distance. (B) Mantel test between Nei’s genetic distance and geographical distance of 12 *Q. liaotungensis* populations. (C) Cluster heat map analysis of 12 *Q. liaotungensis* populations based on 19 climate factors. (D) Mantel test between Nei’s genetic distance and climatic factors of 12 *Q. liaotungensis* populations. bio1: Annual mean temperature; bio2: Mean Diurnal Range; bio3: Isothermality; bio4: temperature seasonality; bio5: max temperature of the warmest month; bio6: min temperature of the coldest month; bio7: annual temperature range; bio8: mean temperature of the wettest quarter; bio9: mean temperature of the driest quarter; bio10: mean temperature of the warmest quarter; bio11: mean temperature of the coldest quarter; bio12: precipitation factors: annual precipitation; bio13: precipitation of wettest month; bio14: precipitation of the driest month; bio15: precipitation seasonality; bio16: precipitation of wettest quarter; bio17: precipitation of driest quarter; bio18: precipitation of warmest quarter; bio19: precipitation of coldest quarter.

**Figure 3:**
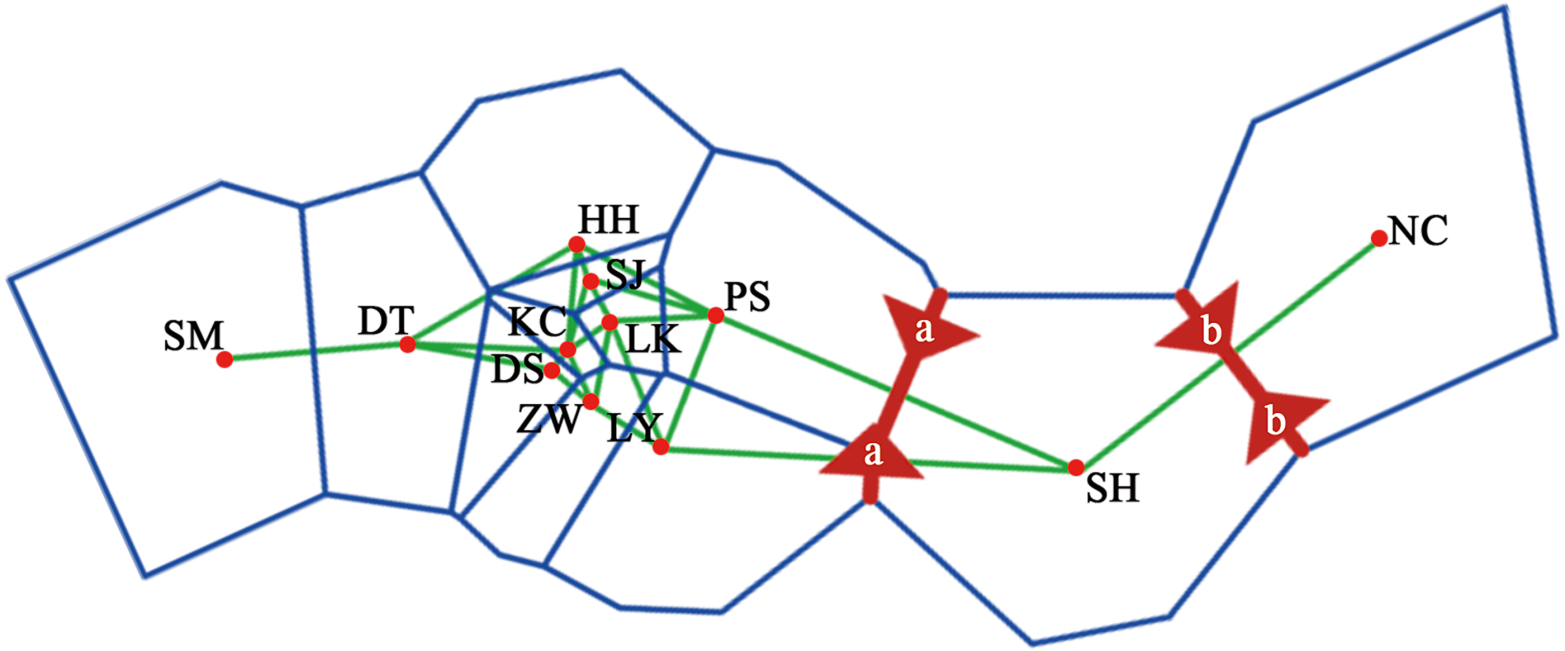
Physical barrier of 12 *Q. liaotungensis* populations predicted by BARRIER (version 2.2).

A dendrogram of 12 *Q. liaotungensis* populations was generated based on Nei’s genetic distances using the Unweighted Pair Group Method with Arithmetic Mean (UPGMA) method (Fig. 2A, Table S2). The *Q. liaotungensis* populations under a coefficient of 0.2 can be divided into 3 clusters: SH population (cluster I), NC population (clusterI I), and other 10 populations (cluster I I I). The result of the Mantel test was significant for Nei’s genetic and geographical distances of 12 *Q. liaotungensis* populations (Fig. 2B), indicating that Nei’s genetic distance was correlated with geographic distance (*r* = 0.638, *p* = 0.028). Analyses of the physical barrier between the 12 *Q. liaotungensis* populations supported the two boundaries shown in Fig. 3. The boundary a, between PS and SH population, isolated the SH population and others 10 *Q. liaotungensis* populations. The boundary b separated NC and SH population.

The cluster heat map of 12 *Q. liaotungensis* populations was constructed based on 19 climatic factors, as shown in Fig. 2C. The results showed that all of *Q. liaotungensis* populations were divided into 3 clusters, and the outcome was highly similar to the cluster analysis based on genetic distance, indicating that the genetic structure of *Q. liaotungensis* may be affected by climatic conditions. However, the result of the Mantel test between Nei’s genetic distance and climatic factors of 12 *Q. liaotungensis* populations was not significant (*r* = 0.544, *p* = 0.120) (Fig. 2D).

**Figure 4:**
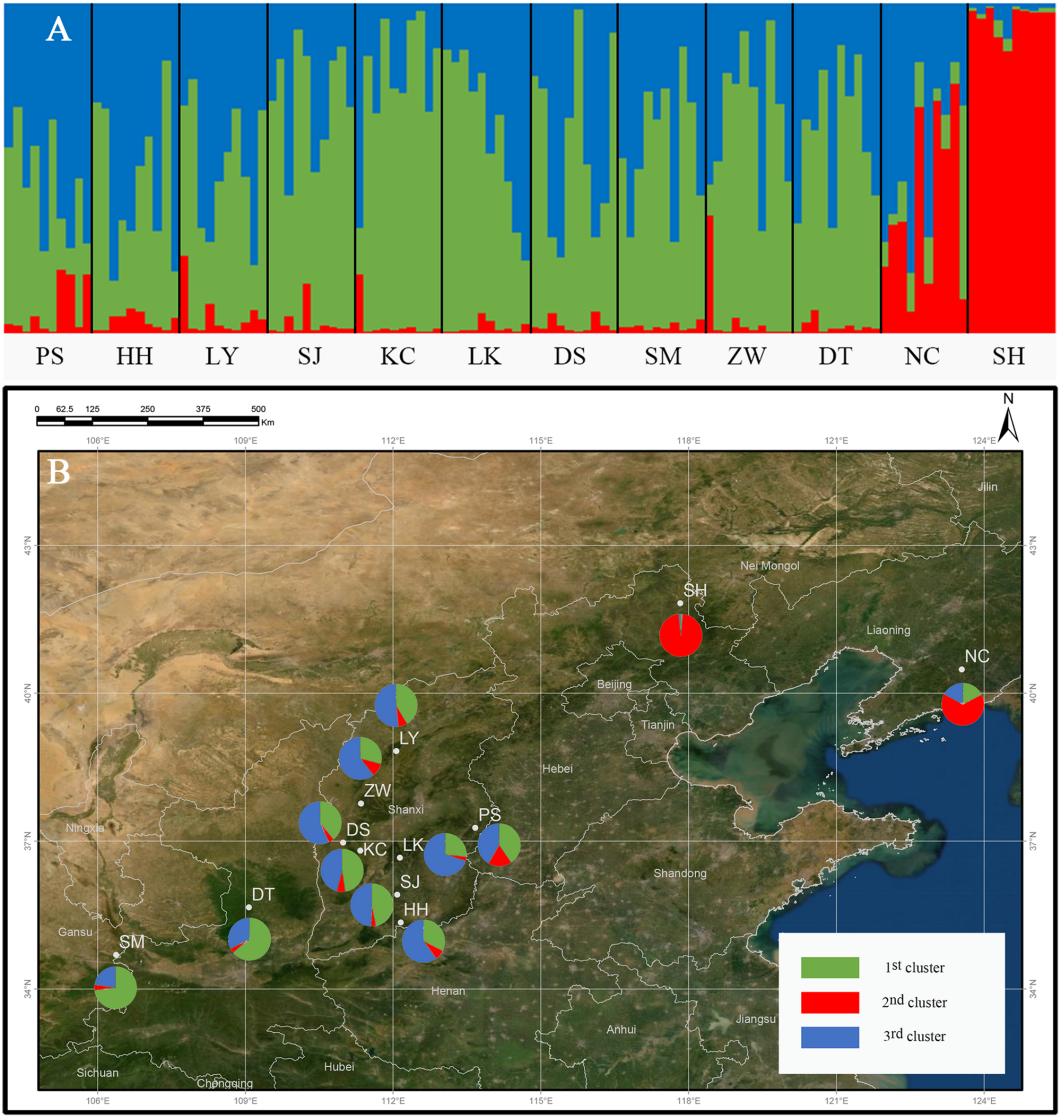
Genetic structure of the population using STRUCTURE analysis. (A) Genetic structure of 12 *Q. liaotungensis* populations (*K* = 3). (B) Proportions of cluster memberships in each of 12 *Q. liaotungensis* populations (*K* = 3). Map credit: ©Esri.

The genetic structure of *Q. liaotungensis* was analyzed with a Bayesian model using STRUCTURE software. The result showed that Δ*K* reached a maximum value when *K* = 3, indicating that 12 *Q. liaotungensis* populations can be divided into 3 subpopulations (Fig. S1). Different colors were used to indicate the proportion of cluster membership in each individual (Fig. 4A), and a visual comparison of the proportion of clusters in each population was performed based on *Q* values. (Fig. 4B). Geographical distribution was relatively straightforward, with the Liaodong Peninsula subpopulation (NC), Bashang Plateau subpopulation (SH), and Loess Plateau subpopulation (other 10 populations) exhibiting significant differences. For the Loess Plateau subpopulation, the first cluster (green) and the third cluster (blue) had a more significant proportion, whereas the second cluster (red) had a smaller proportion. Meanwhile, the second cluster (red) had a larger proportion in the Bashang Plateau subpopulation and the Liaodong Peninsula subpopulation.

In addition, we independently investigated the genetic differentiation and gene flow among 3 subpopulations, and found that the higher values of *F*st. The *F*st value between Bashang plateau subpopulation (SH) and Liaodong peninsula subpopulation (NC) was 0.054, and between Bashang plateau subpopulation and Loess plateau subpopulation (other 10 populations) was 0.068, indicating a moderate level of genetic differentiation among 3 subpopulations. Meanwhile, we have investigated gene flow (*Nm*) among 3 subpopulations. The pair-wise value of *Nm* among 3 subpopulations was 3.41 (Bashang Plateau vs. Loess Plateau), 7.44 (Liaodong Peninsula vs. Loess Plateau), and 4.38 (Liaodong Peninsula vs. Bashang Plateau), respectively.

## Discussion

### Genetic diversity

At the species level, genetic diversity is usually related to the life cycle, geographic range, mating system, migration, and balancing selection (Gaudeul, Taberlet & Till-Bottraud, 2000; Hamrick & Godt, 1990; Hellmann & Pineda-Krch, 2007; Li et al., 2008). Generally, the widely distributed long-lived Quercus species exhibit abundant genetic diversity. In this study, *Q. liaotungensis* populations showed a high level of genetic diversity, especially concerning heterozygosity (*H*e = 0.801), which was higher than that of other Quercus species, such as summer oak (*Q. robur*) (*H*e = 0.764) (Sullivan et al., 2013), sawtooth oak (*Q. acutissima*) (*H*e = 0.660) (Zhang et al., 2013), and Mongolian oak (*Q. mongolica*) (*H*e = 0.630) (Ueno & Tsumura, 2008). On the one hand, high genetic diversity may be attributed to the biological characteristics of *Q. liaotungensis*. The geographical boundary of *Q. liaotungensis* spans from the northern slope of the Qinling Mountains to the southern slope of the Changbai Mountains (Li et al., 2012). The widespread distribution and heterogeneous habitats may lead to high levels of genetic diversity because abundant genotypes can enhance the ability of species to respond to different environments. On the other hand, the mating system with unique outcrossing and long-distance gene flow model can also strengthen the genetic diversity. In addition, the genetic diversity of *Q. liaotungensis* (*H*e = 0.801) in this report was higher than that of previous report (*H*e = 0.754) (Qin et al., 2012). The main reason could be the different sampling sites and that the number of samples in this study was more than that in previous studies. The other reason could be the various types of climatic environments and soil conditions in these sampling sites (Yang et al., 2018).

At the population level, the NC population had the highest genetic diversity out of the 12 *Q. liaotungensis* populations. The NC population is located in the Liaodong Peninsula of Northeast China, where the ecological niche overlap for *Q. liaotungensis* and *Q. mongolica* species. Therefore, the natural hybridization of the 2 species could lead to gene introgression and show a much larger genetic diversity than the other population*s*. The SH population had the lowest genetic diversity and significantly deviated from the HWP. Fewer individuals existed in secondary forests with a patchy distribution of *Q. liaotungensis*, thereby causing this phenomenon.

### Genetic differentiation and genetic structure

Compared to the herbaceous plants, the woody plants have lower genetic differentiation among 12 *Q. liaotungensis* populations and higher genetic variation within 12 *Q. liaotungensis* populations. This difference could be attributed to high outcrossing rates, reproduction, wind-pollination, and acorn transport (Radu et al., 2014). The level of genetic differentiation among *Q. liaotungensis* population was low (*F*st = 0.033), and the result was consistent with the recent research on Quercus species, such as holm oak (*Q. ile* x) (*F*st = 0.018) (Guzmán et al., 2015) and cork oak (*Q. suber*) (*F*st = 0.017) (Coelho et al., 2006). Moreover, gene flow and genetic drift have a strong influence on the genetic differentiation of different populations (Schaal et al., 1998). In the *Q. liaotungensis* species, wind-pollination, outcrossing, long-distance pollen dispersal, spreading of acorns by animals, and relatively high gene flow (*N*m = 11.286) may limit genetic drift and reduce genetic differentiation.

The genetic structure of *Q. liaotungensis* is divided into 3 subpopulations with the Bayesian model, which is consistent with the results of the cluster analysis based on Nei’s genetic distance. Furthermore, the results of the Mantel test showed that the genetic distance had a significantly positive correlation with the geographic distance (*r* = 0.638, *p* = 0.028), indicating clear isolation by distance (IBD) among the investigated *Q. liaotungensis* populations. A larger distance from 35.89 km (between KC and DS population) to 1,642.82 km (between NC and SM population) based on the latitude and longitude may lead to a more significant genetic differentiation. In addition, two boundaries exist in 3 groups of 12 *Q. liaotungensis* populations. The boundary a, isolated the 10 *Q. liaotungensis* populations from Loess Plateau in China by Taihang Mountains. The boundary b, corresponding to the Liao River, and separated the NC population from the Liaodong Peninsula and SH population from Bashang Plateau. Therefore, the geographical distance and physical barriers jointly affected the genetic differentiation among *Q. liaotungensis* populations. Additionally, the genetic differentiation of marginal populations of the *Q. liaotungensis* was higher than that of the central populations in China (Petit, Mousadik & Pons, 1998). This genetic differentiation among populations could be attributed to habitat destruction in certain areas, which is affected by climate change and illegal logging.

The genetic structure formation not only depends on geographical isolation but also affected by the changes of distribution and size mediated by climatic conditions (Ortego et al., 2012). Studies have shown that in addition to the influence of geographical distance, the ecological conditions exert a significant influence on the genetic variation patterns of *Fraxinus mandshurica* and *Elymus athericus* (Bockelmann et al., 2003; Temunovic et al., 2012). *Q. liaotungensis* has adapted to different ecological niches with unique temperature and precipitation seasons, leading to high genetic differentiation within populations (Li et al., 2012; Yang et al., 2018). In this study, the cluster analysis based on 19 climatic factors revealed obvious differences between the climatic types of the 3 clusters. Climate differences caused by environmental heterogeneity may promote the formation of the genetic structure of *Q. liaotungensis*, and this result was similar to that of the study by Yang et al. The main reason as follow, firstly, *Q. liaotungensis* populations split before the Last Glacial Maximum (LGM) and have adapted to distinct ecological niches with unique temperature and precipitation seasonality, resulting in high genetic differentiation. Interestingly, the NC and SH population have higher precipitation (bio12, bio13, bio14, bio16, bio17, bio18, and bio19) and temperature differences (bio4 and bio7) when compared to other populations, respectively, and these two populations had been separately divided into 2 distinguished clusters. Secondly, *Q. liaotungensis* populations showed a dynamic expansion decline trend during the Quaternary climatic oscillations and increased genetic differentiation between central and peripheral populations. Hence, we can conclude that climatic conditions exert an important influence on the genetic structure of *Q. liaotungensis*.

In addition, the formation of genetic structure is related to hybridization and gene infiltration. Current studies have shown that the hybridization of *Q. liaotungensis* and *Q. mongolica* occurs in areas of ecological niche overlap. The hybrid traits, such as all of the differentiated leaf and reproductive traits, tend to those of *Q. liaotungensis* (Wei et al., 2015). The populations located around the Changbai Mountains in Northeast China retained the characteristics of *Q. liaotungensis,* but introduced alleles from *Q. mongolica* (Zeng et al., 2011). This study found that the NC and SH populations in the Liaodong Peninsula and the Bashang Plateau are located in the overlapping niche areas of *Q. liaotungensis* and *Q. mongolica*, respectively. Furthermore, the structural analysis showed the second cluster (red) of pie charts to be larger than the other groups. In addition, studies have shown that interspecific hybridization frequently occurs in the closely related species of Quercus (Gonzĺez-Rodríguez & Oyama, 2005; Lagache et al., 2013) causing gene introgression in the hybrid zones (Wei et al., 2015). The above evidence suggests that the *Q. liaotungensis* population in the niche overlap region may be a natural phenomenon of hybrid and gene introgression among the two species.

### Conservation strategies of *Q. liaotungensis*

The germplasm resources of *Q. liaotungensis* suffered a continuous decline because of frequent human activities and weak natural regeneration. To prevent a decrease in population size and loss of genetic diversity, the protection and management of *Q. liaotungensis* should be strengthened. Our results showed that the genetic variation pattern of the *Q. liaotungensis* population was related to geographical isolation, physical barrier, climatic types, and natural hybridization. For *in situ* protection, establishing at least one respective conservation unit in 3 different areas (Liaodong Peninsula, Bashang Plateau, and Loess Plateau) is required. Meanwhile, seeds and scions should be collected to establish germplasm pools for *ex-situ* protection. Because the genetic variation exists mainly within the populations, we need to collect as many families and individuals as possible at each population. In addition, active management, such as the establishment of seed forests and artificial promotion of natural regeneration, is necessary.

## Conclusions

In this study, we evaluated the genetic diversity and structure of 12 *Q. liaotungensis* populations in China using 19 SSR primer pairs. The *Q. liaotungensis* population presents a relatively high genetic diversity and low genetic differentiation, with the majority of variations occurring mainly within the population. The STRUCTURE analysis shows that the genetic structure of *Q. liaotungensis* populations can be divided into three subpopulations. The Mantel test reveals a positive correlation between Nei’s genetic distance and geographic distance, and two boundaries were found among the 12 *Q. liaotungensis* populations by the Barrier analysis, indicating that *Q. liaotungensis* populations have been isolated by distance and physical barrier. In addition, climatic conditions and natural hybridization influence the genetic structure of *Q. liaotungensis* populations. All together, the findings of this study will play an essential role in the conservation and genetic improvement of *Q. liaotungensis*.

##  Supplemental Information

10.7717/peerj.10922/supp-1 Supplemental Information 1 Relationships between the number of clusters (*K*) and the corresponding Delta K statistics based on STRUCTURE analysis (*K* = 1 − 10)

10.7717/peerj.10922/supp-2 Supplemental Information 2 Analysis of molecular variance (AMOVA) among and within 12 *Q.liaotungensis* populations using 19 SSR markers

10.7717/peerj.10922/supp-3 Supplemental Information 3 The pairwise genetic distance (*GD*) values among 12 *Q.liaotungensis* populations using 19 SSR markers

10.7717/peerj.10922/supp-4 Supplemental Information 4 The pairwise genetic differentiation (*F*st) values among 12 *Q.liaotungensis* populations using 19 SSR markers

10.7717/peerj.10922/supp-5 Supplemental Information 5 Raw allele data

10.7717/peerj.10922/supp-6 Supplemental Information 6 Raw data

## Additional Information and Declarations

### Data Availability

The following information was supplied regarding data availability: Raw measurements are available in the Supplemental Files.
