# Supplementary figures and images for: Unraveling the genetic diversity and structure of Quercus liaotungensis population through analysis of microsatellite markers
Source: PeerJ. 2021 Apr 14;9:e10922. doi: 10.7717/peerj.10922 (PMC8052960; doi:10.7717/peerj.10922)

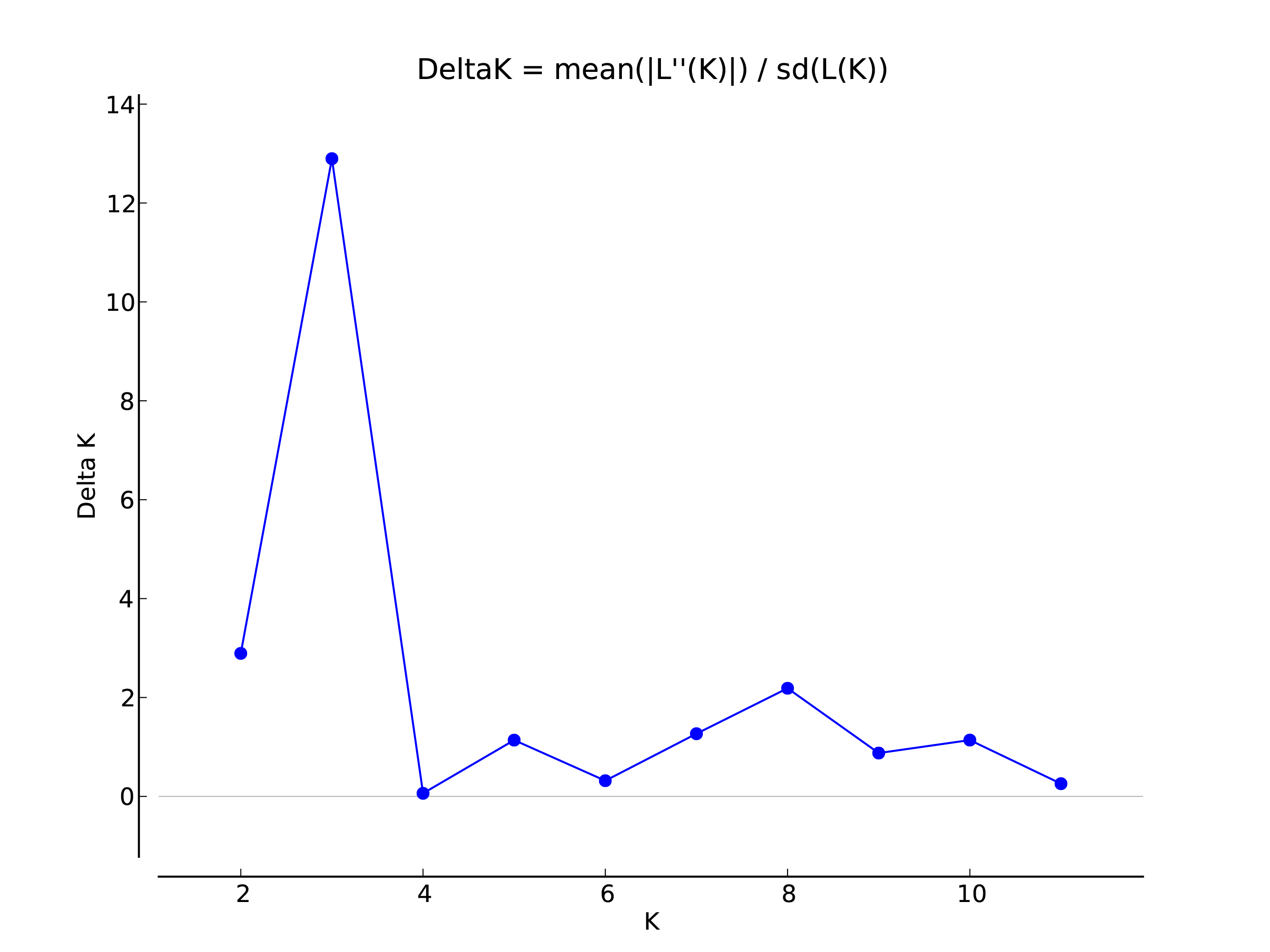

Supplement: Supplemental Information 1 [file peerj-09-10922-s001.png]
