# Supplementary material for: Unraveling the genetic diversity and structure of Quercus liaotungensis population through analysis of microsatellite markers
Source: PeerJ. 2021 Apr 14;9:e10922. doi: 10.7717/peerj.10922 (PMC8052960; doi:10.7717/peerj.10922)
Supplement: Supplemental Information 2 [file peerj-09-10922-s002.docx]

**Table S1**. Analysis of molecular variance (AMOVA) among and within 12 *Q.liaotungensis* populations using 19 SSR markers

| Source of variation | Degree of  freedom | Sum of  squares | Mean square | Variance  component | Percentage of  variation (%) |
| --- | --- | --- | --- | --- | --- |
| Among Populations | 11 | 293.067 | 26.642 | 0.984 | 5.5% |
| Within Populations | 108 | 1814.500 | 16.801 | 16.801 | 94.5% |
| Total | 119 | 2107.567 |  | 17.785 | 100% |
