# Supplementary material for: Unraveling the genetic diversity and structure of Quercus liaotungensis population through analysis of microsatellite markers
Source: PeerJ. 2021 Apr 14;9:e10922. doi: 10.7717/peerj.10922 (PMC8052960; doi:10.7717/peerj.10922)
Supplement: Supplemental Information 3 [file peerj-09-10922-s003.docx]

**Table S2.** The pairwise genetic distance (*GD*) *va*lues among 12 *Q.liaotungensis* populations using 19 SSR markers

| Population | PS | HH | LY | SJ | KC | LK | DS | SM | ZW | DT | NC | SH |
| --- | --- | --- | --- | --- | --- | --- | --- | --- | --- | --- | --- | --- |
| PS | 0.0000 |  |  |  |  |  |  |  |  |  |  |  |
| HH | 0.1130 | 0.0000 |  |  |  |  |  |  |  |  |  |  |
| LY | 0.1175 | 0.1606 | 0.0000 |  |  |  |  |  |  |  |  |  |
| SJ | 0.1732 | 0.1501 | 0.1548 | 0.0000 |  |  |  |  |  |  |  |  |
| KC | 0.1577 | 0.1408 | 0.1344 | 0.0891 | 0.0000 |  |  |  |  |  |  |  |
| LK | 0.2212 | 0.1107 | 0.1559 | 0.1298 | 0.1237 | 0.0000 |  |  |  |  |  |  |
| DS | 0.1435 | 0.1355 | 0.1980 | 0.1233 | 0.1235 | 0.1521 | 0.0000 |  |  |  |  |  |
| SM | 0.1637 | 0.1221 | 0.2655 | 0.1998 | 0.1724 | 0.1850 | 0.1581 | 0.0000 |  |  |  |  |
| ZW | 0.1609 | 0.1203 | 0.1618 | 0.0926 | 0.0942 | 0.0783 | 0.0719 | 0.1488 | 0.0000 |  |  |  |
| DT | 0.1551 | 0.1616 | 0.1754 | 0.1790 | 0.1166 | 0.2233 | 0.1574 | 0.2076 | 0.1340 | 0.0000 |  |  |
| NC | 0.2319 | 0.2783 | 0.2754 | 0.2816 | 0.3573 | 0.3301 | 0.2903 | 0.3397 | 0.2734 | 0.3168 | 0.0000 |  |
| SH | 0.5209 | 0.5403 | 0.4798 | 0.6212 | 0.7080 | 0.6725 | 0.6334 | 0.6281 | 0.6026 | 0.6335 | 0.3332 | 0.0000 |
