# Supplementary material for: Unraveling the genetic diversity and structure of Quercus liaotungensis population through analysis of microsatellite markers
Source: PeerJ. 2021 Apr 14;9:e10922. doi: 10.7717/peerj.10922 (PMC8052960; doi:10.7717/peerj.10922)
Supplement: Supplemental Information 4 [file peerj-09-10922-s004.docx]

**Table S3.** The pairwise genetic differentiation (*F*st) values among 12 *Q.liaotungensis* populations using 19 SSR markers

| Population | PS | HH | LY | SJ | KC | LK | DS | SM | ZW | DT | NC | SH |
| --- | --- | --- | --- | --- | --- | --- | --- | --- | --- | --- | --- | --- |
| PS | 0.0000 |  |  |  |  |  |  |  |  |  |  |  |
| HH | 0.0002 | 0.0000 |  |  |  |  |  |  |  |  |  |  |
| LY | 0.0003 | 0.0096 | 0.0000 |  |  |  |  |  |  |  |  |  |
| SJ | 0.0179 | 0.0107 | 0.0105 | 0.0000 |  |  |  |  |  |  |  |  |
| KC | 0.0183 | 0.0127 | 0.0098 | 0.0000 | 0.0000 |  |  |  |  |  |  |  |
| LK | 0.0309 | 0.0005 | 0.0117 | 0.0071 | 0.0091 | 0.0000 |  |  |  |  |  |  |
| DS | 0.0111 | 0.0078 | 0.0217 | 0.0056 | 0.0096 | 0.0147 | 0.0000 |  |  |  |  |  |
| SM | 0.0148 | 0.0029 | 0.0354 | 0.0248 | 0.0218 | 0.0218 | 0.0147 | 0.0000 |  |  |  |  |
| ZW | 0.0105 | 0.0000 | 0.0075 | 0.0000 | 0.0000 | 0.0000 | 0.0000 | 0.0074 | 0.0000 |  |  |  |
| DT | 0.0151 | 0.0157 | 0.0177 | 0.0219 | 0.0083 | 0.0344 | 0.0170 | 0.0287 | 0.0060 | 0.0000 |  |  |
| NC | 0.0224 | 0.0309 | 0.0259 | 0.0352 | 0.0550 | 0.0457 | 0.0374 | 0.0457 | 0.0270 | 0.0440 | 0.0000 |  |
| SH | 0.0939 | 0.0972 | 0.0824 | 0.1142 | 0.1326 | 0.1239 | 0.1160 | 0.1148 | 0.1031 | 0.1178 | 0.0522 | 0.0000 |
